# Effect of introducing frogs and fish on soil phosphorus availability dynamics and their relationship with rice yield in paddy fields

**DOI:** 10.1038/s41598-019-56644-z

**Published:** 2020-01-08

**Authors:** Kaimiao Lin, Jianping Wu

**Affiliations:** 1 Department of Tourism Management, Wuyi University, Nanping city, Fujian, China; 2 School of Geographical Sciences, Fujian Normal University, Fuzhou city, Fujian, China; 3 Hong-Jian Science and Technology Farm, Nanping city, Fujian, China

**Keywords:** Agroecology, Element cycles

## Abstract

The long-term impacts of introducing frogs and fish on rice yield and soil P availability are largely underestimated and undervalued. A 9-year field experiment compared the soil phosphorus fraction dynamics and their relationship with rice yield in rice-frog-fish (RFF) cultures, rice-fish (RF) cultures and rice-only (RO) cultures in southeastern China paddy fields. The yields in the RFF and RF cultures were notably higher than those in the RO culture, by 22.1% and 6.8%, respectively. Soil total P ranged from 345.5 to 385.6 mg kg^−1^ among all the farming systems, with the smallest amount found in the RO culture. There were only slight changes in the distribution of soil phosphorus fractions with time. The concentrations of NaHCO_3_-Pi and NaHCO_3_-Po were significantly higher in the RFF and RF cultures compared with those in the RO culture, and most of the NaOH-Pi and NaOH-Po fractions were greater in the RFF and RF cultures compared with those in the RO culture. The rice grain yield was significantly correlated with labile P and slowly cycling P. Introducing frogs and fish might be useful for increasing soil active P supplies and meeting rice nutrient requirements. This study concluded that RFF is the best practice for improving rice grain yields and soil fertility in paddy fields.

## Introduction

The rice-fish farming system, which is an integrated agro-ecosystem practice, is believed to be an effective method for increasing food production in fields through ecological agriculture^1^. It is a practice that can be found in many countries across Asia but that is common in southeastern China^2^. Many different types of agro-ecological methods have been developed based on the water requirements for growing rice, which include the simultaneous production of rice-fish, rice-crab, rice-duck and rice-prawn-fish^3–5^. The rice-fish farming system has a long history of more than 1200 years in some parts of China. As such, local farmers have been able to create a diverse range of cultivation patterns and techniques^6,7^. However, the rice-frog-fish farming system is a recent practice in South and Southeast Asia, and its long-term benefits are still largely unknown.

The benefits of the rice-frog-fish farming system are not limited to the additional production of frogs and fish. It is assumed that the frogs and fish serve as a form of weed and pest control in the paddy fields^8^. Biological control is the dominant method of integrated pest management implemented by an agricultural company in the suburbs of Shanghai^9^. The rice-frog-fish farming system is successful in sustaining the ecological balance in paddy fields; this system not only increases the grain yield but also is a more sustainable farming system compared with that of traditional agriculture. The frog and fish excrement also has a fertilizing effect, which increases the nutrients that are available to the rice crops^10,11^ This system may also improve the soil fertility and conservation of local soil and water resources in subtropical rice fields^10^.

The production of rice, one of the main food crops in developing countries, has increased in recent years, and most fields have been planted in subtropical upland rice paddy soil^12^. However, the production of rice is largely limited due to the phosphorus deficiency in many soils in subtropical and tropical regions^13,14^. Plant growth is highly dependent on soil phosphorus, which is a vital limiting nutrient. This is particularly true in the subtropical and tropical regions, where the primary production of various ecosystems is regarded as P limited but not N limited^15^. To date, few studies have focused on soil P fractions and available P in response to rice-frog-fish cultures and rice-fish cultures. Previous studies have reported contrasting results concerning the effects of different integrated agro-ecological cultures during short-term experimental work. Introducing frogs in the rice-growing season could increase the contents of soil total P and available P in paddy fields^8^. However, Yi *et al*. reported that soil-available phosphorus in rice-fish cultures was significantly decreased^16^. Therefore, long-term experimental work has examined the influence of introducing frogs or fish on rice productivity and soil P availability and dynamics in paddy fields. However, little is known about the dynamics of soil P fractions in response to different ecological agricultural practices in southeastern China paddy fields. The chemical characteristics of different P forms in soil, which are derived using the sequential extraction method developed by Hedley^17^, were used to determine the ease of phosphorus absorption by crops. It is essential to understand how conventional intensive farming systems and ecological agriculture systems affect the soil phosphorus availability and its various forms, as farming practices can affect plant available and refractory forms of soil phosphorus. The potential solubility of phosphorus may indicate its role as a pollutant, as it can leach into the soil solution and enter the groundwater and surface water. Understanding the differences in the soil phosphorus dynamics between different farming systems is critical for the examination of ecological and nutritional balances.

A sequential extraction procedure was described as a popular concept by Walker and Syers in 1976. It was then modified by Tiessen and Moir in 1993 and has been extensively used to assess the available and refractory forms of soil phosphorus^18^. Applications of sequential phosphorus fractionation are common in cultivation^17^ and land use and management^19^. However, there has been limited information on upland rice paddy fields. The phosphorus chemistry in upland rice soils is unique relative to that of other soils due to wetting and drying cycles. While these processes have been shown to affect the short-term phosphorus dynamics^20^, the long-term effects on P dynamics remain unknown. Several studies that use sequential phosphorus fractionation procedures have investigated how different farming systems have affected the phosphorus availability within soil^21^. However, previous research has shown that the impacts of different farming systems on soil phosphorus dynamics were short-lived and restricted to specific locations.

This study aims to (i) determine whether rice-frog-fish farming systems in paddy fields increased rice yields with increased time and (ii) evaluate the response of phosphorus fractions and phosphorus availability in rice-frog-fish farming systems compared to that in rice-fish and rice-only farming systems.

## Results

### Rice grain yield under the different farming systems

**Figure 1:**
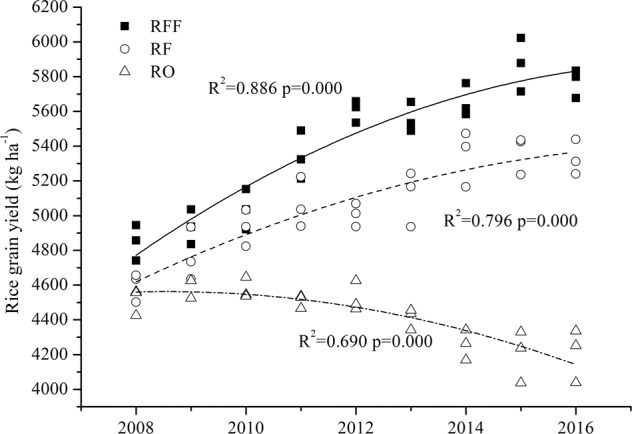
Rice grain yield response to the rice-frog-fish (RFF) culture, rice-fish (RF) culture and rice-only (RO) culture from 2008 to 2016.

According to a survey of subtropical paddy soil from 2008 to 2016, the highest mean annual grain yield of rice per hectare was found in the RFF (5402.54 kg ha^−1^), followed by the RF (5058.31 kg ha^−1^) and the RO farming systems (4422.31 kg ha^−1^) (Table 1). This study showed that during the 9-year field experiment, grain yields in the RFF and RF farming systems were notably higher, by 22.1% and 6.8%, respectively, than that in the RO. The greatest RFF yield was 5871.85 kg ha^−1^ in 2009. There was a significant difference (*P* < 0.05) in rice yield between the different farming systems from 2008 to 2016 (Table 1). The polynomial function showed that the rice grain yield of the RFF and RF farms increased rapidly from 2008 to 2013, then the rate of increase gradually smoothed out, but the rice grain yield of the RO farm declined gradually from 2008 to 2016 (Fig. 1).

**Table 1:** Mean ± SD for rice grain yield parameters in the rice-frog-fish, rice-fish, and rice-only farming systems.

Year	Treatment (kg ha^−1^)		
	Rice-only	Rice-fish	Rice-frog-fish
2008	4515.28 ± 77.2c	4597.26 ± 83.9b	4847.98 ± 102.5a
2009	4592.02 ± 57.7c	4767.89 ± 152.8a	4935.60 ± 100.0a
2010	4575.91 ± 61.1c	4930.64 ± 104.6b	5036.95 ± 116.1a
2011	4510.47 ± 39.2b	5066.49 ± 144.2b	5341.80 ± 139.2a
2012	4526.36 ± 88.8c	5006.30 ± 66.6b	5605.85 ± 63.4a
2013	4411.00 ± 60.2c	5115.15 ± 159.4b	5558.18 ± 85.7a
2014	4259.06 ± 86.8c	5345.15 ± 159.5b	5654.85 ± 94.5a
2015	4201.4 ± 149.5c	5365.96 ± 112.4b	5871.85 ± 153.9a
2016	4209.27 ± 153.6c	5329.98 ± 100.7b	5769.85 ± 81.9a
Total	4422.31 ± 170.9c	5058.31 ± 276.7b	5402.54 ± 374.7a

Different letters indicate significantly different rice grain yields each year between rice-frog-fish, rice-fish and rice-only farming systems at p < 0.05.

### Soil phosphorus fractions in response to different farming systems

**Figure 2:**
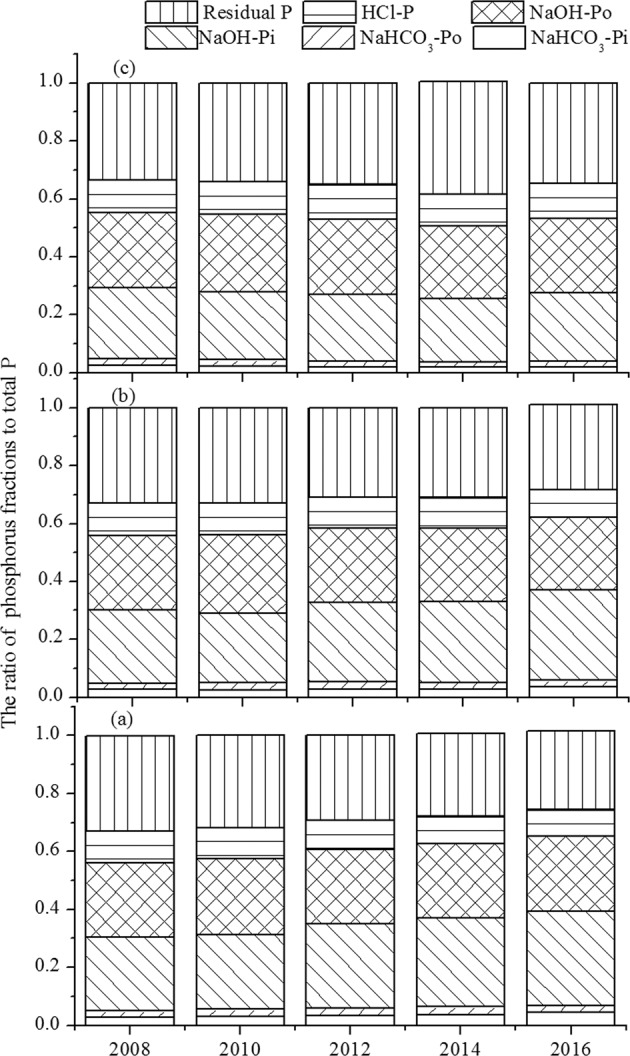
Trends of P fractions (% of total P) of different lability over a 9-year field experiment. (**a**) RO culture; (**b**) RFF culture; (**c**) RF culture.

The changing trend of different soil P fractions in the three agro-ecosystems during the 9-year experiment is shown in Fig. 2. Overall, there were few changes in the percent of various P fractions. The labile-Pi (NaHCO_3_-Pi) and labile-Po (NaHCO_3_-Po) forms constitute the smallest phosphorus fractions, with less than 3.5% of the total phosphorus and ranging from 7.5 to 12.1 mg kg^−1^ and 7.3 to 9.1 mg kg^−1^, respectively. The residual P fraction, which predominated the soil phosphorus, ranged from 108.9 to 128.9 mg kg^−1^ and constituted 32.6% to 33.5% of the soil total P. This was followed by the slowly cycling inorganic P (81.0–95.7 mg kg^−1^ and 23.1–26.5% of total P) and slowly cycling organic P (92.8–107.6 mg kg^−1^ and 24.0–27.3% of total P).

**Figure 3:**
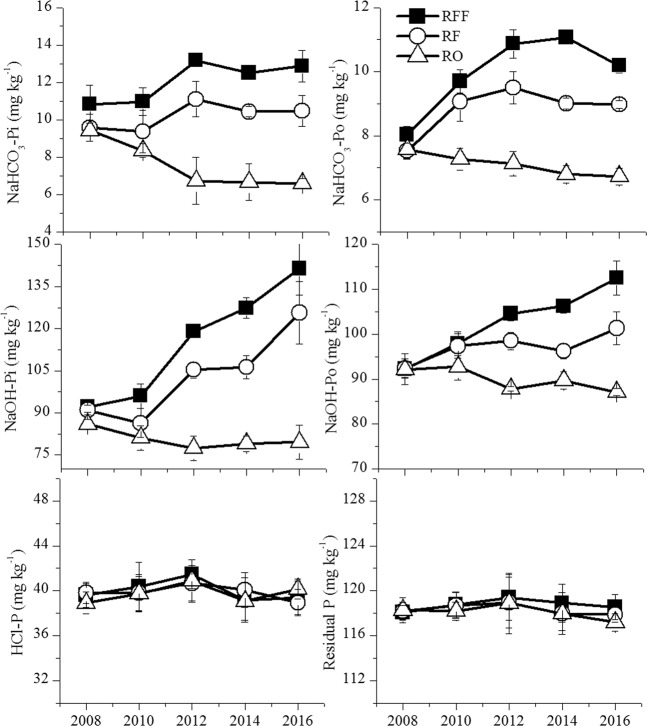
Response of soil phosphorus fractions to the 9-year rice yield of the rice-frog-fish (RFF) culture, rice-fish (RF) culture and rice-only (RO) culture. (**a**) NaHCO_3_-Pi, (**b**) NaHCO_3_-Po, (**c**) NaOH-Pi, (**d**) NaOH-Po, (**e**) HCl-Pi, (**f**) residual P. Error bars indicate standard errors (n = 3).

The concentrations of NaHCO_3_-Pi and NaHCO_3_-Po were significantly higher in the RFF and RF treatments compared with those in the RO treatment for most years during the 9-year field experiment (Fig. 3a,b), and most of the NaOH-extractable Pi and Po fractions were greater in the RFF and RF treatments compared with those in the RO treatment. Nonetheless, no consistent differences were found in the HCl-P and residual P among RFF, RF, and RO (Fig. 3c,d). Changes in the phosphorus fractions following the planting time were readily evident (Table 2) but differed among the different farming systems. With the exception of the HCl-P and residual P fractions, all the sequential extraction phosphorus in the RFF and RF treatments gradually increased from 2008 to 2016 but decreased in the RO culture during the 9-year experimental period (Fig. 3e,f). There were no significant changes in the HCl-P and residual P in the different farming systems, but the differences in the NaOH-extractable P and NaHCO_3_-extractable P fractions were highly significant between the different farming systems (Table 2).

**Table 2:** *P*-values for the significant differences of a paired *t*-test for time (2008, 2010, 2012, 2014, and 2016) and farming systems (rice-frog-fish (RFF) culture, rice-fish (RF) culture and rice-only (RO) culture).

Pool	Time	Farming system
NaHCO_3_-Pi	0.884	<0.001
NaHCO_3_-Po	0.180	<0.001
NaOH-Pi	0.002	<0.001
NaOH-Po	0.032	0.001
HCl-P	0.856	0.765
Residual P^a^	0.709	0.428
Labile P^b^	0.446	<0.001
Slowly cycling P^c^	0.002	<0.001
Total P^d^	0.007	<0.001

^a^Occluded P.

^b^Sum of NaHCO_3_-Pi and NaHCO_3-_Po.

^c^Sum of NaOH-Pi, NaOH-Po and HCl-P.

^d^Independent total P analysis.

**Figure 4:**
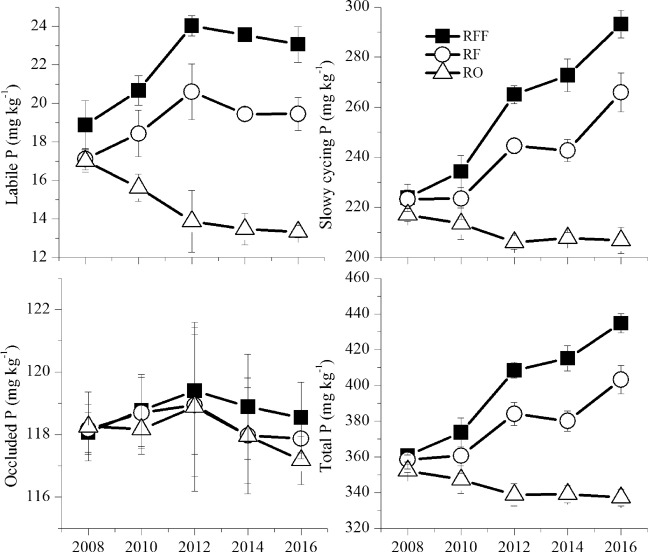
Response of soil labile P, slowly cycling P, occluded P and total P to the 9-year rice yield of the rice-frog-fish (RFF) culture, rice-fish (RF) culture and rice-only (RO) culture. (**a**) Labile P = NaHCO_3_-Pi + NaHCO_3_-Po, (**b**) slowly cycling P = NaOH-Pi + NaOH-Po + HCl-P, (**c**) occluded P = residual P, (**d**) total P. Error bars indicate the standard errors (n = 3).

Labile P (NaHCO_3_-Pi + NaHCO_3_) declined over the 9-year field cultivation period in the RO system but significantly increased in the RFF and RF systems (Fig. 4a). There was a considerable increase over time in the concentration of slowly cycling P (NaOH-Pi + NaOH-Po + HCl-P) in all the farming systems, especially in the RFF system (Fig. 4b). However, there were no consistent differences in residual P between all three farming systems (Fig. 4c). The soil total P ranged from 345.5 to 385.6 mg kg^−1^ among all the farming systems, with the smallest amount found in the RO system (Fig. 4d). There were significant differences in most labile P and slowly cycling P fractions between the RFF, RF and RO treatments from 2008 to 2016 (Table 2).

### Relationships between soil P fractions and rice grain yield

The visible trend in the variation between soil phosphorus fractions and the rice grain yield in the RFF, RF and RO farming systems are shown in Table 3. No significant differences were found in any of the phosphorus fractions in the RO treatment. In the RFF and RF treatments, there was a significant increase in all the P fractions in the rice grain yields, including labile P and slowly cycling P, but no significant difference was observed with the occluded P. There was a significant relationship between the rice grain yield and all the phosphorus fractions, but even more important was the highly significant relationship between the rice grain yield and the soil labile P. This relationship showed that soil labile P in the subtropical upland paddy soil is very important for rice yield.

**Table 3:** Pearson’s correlation coefficients between soil phosphorus fractions and rice grain yield in different farming systems: rice-frog-fish (RFF) culture, rice-fish (RF) culture and rice-only (RO) culture.

Soil P fraction	RO	RF	RFF
Labile P^b^	0.518	0.805**	0.803**
Slowly cycling P^c^	−0.163	0.609*	0.573*
Occluded P^a^	0.170	0.144	0.139
TP^d^	0.132	0.210	0.389

^a^Residual P.

^b^Sum of NaHCO_3_-Pi and NaHCO_3_-Po.

^c^Sum of NaOH-Pi, NaOH-Po and HCL-P.

^d^Independent total P analysis.

*P = 0.05, **P = 0.01 (n = 3).

## Discussion

The traditional rice-fish farming system, which is a “globally important agricultural heritage system” (GIAHS) according to the Food and Agriculture Organization (FAO), has been assumed to have a better overall resource-use efficiency than that of conventional agriculture. Reports have shown that the ecological legacy of the integration of rice and fish in traditional agricultural systems may lead to the development of innovative sustainable agriculture. The principles of symbiosis, mutual benefits and management practices for rice-fish agro-ecological systems have been well documented in Egypt, Thailand, Vietnam, Bangladesh, and other developing countries^22^. The symbiotic benefits of this system are as follows: biological control agents remove weeds and pests that are harmful to rice and increase the turnover of organically bound nutrients^23^, and the rice provides shade, which reduces the water temperature in hot weather, and supplemental food for the fish when planthoppers and other herbivorous insects are preyed upon by fish and frogs and then fall into the water^24^. However, the concurrent cultivation of frogs and fish in rice paddies is a recent practice in South and Southeast Asia and has yet to be well documented. The key benefits of frog culture in rice paddy fields are the minimization of human activities, which can interfere with rice growth, and the improvement of the rice grain yield.

According to Mishra^12,25^ and Mohanty^12,25^, the rice grain yields increased by 8–15% in the fish-rice culture. In our 9-year field experiment, the average rice yields increased by 22.1% in the RFF farming system and 6.8% in the RF farming system compared to the yields of the RO farming system. However, a 6-year survey by Xie^26^ showed that the rice yields between RF and RO farming systems did not differ. This is probably due to the application of pesticides to and large amounts of manure or chemical fertilizer in the RF and RO farming systems that were surveyed in Xie’s study. However, the rice-fish system requires the use of little to no pesticides, which can be harmful to the fish^27^. Therefore, no pesticides were applied, and low amounts of manure or chemical fertilizer were used in the management of rice growth. Previous studies have reported that the production of average annual rice grain yields for RFF, RF and RO were 5.4, 5.0 and 4.4 t ha^−1^, respectively. Many factors, including the variability and change in climate, pest incidence, crop management, and fertilizer rate, result in marked variations in rice yield^26^. In the long run, the rice-frog-fish and rice-fish cultures in this field experiment significantly increased rice grain yield compared to that of the rice-only culture. The increase in rice yield could be attributed to the movement of the frogs and fish, which helped to increase dissolved oxygen levels^28^ and perturbate soil nutrients^7^. The frogs and fish feed on aquatic insects, plankton and aquatic weeds, which compete with rice for nutrients and energy^29^. This may not only reduce the impact of pests and diseases but also promote crop growth and increase yield. Frog and fish dung may enhance soil organic matter^30^ and other nutrients^23^ to naturally fertilize crops.

Soil acidity and high weathering in the subtropical and tropical paddy soil changed the availability of phosphorus, resulting in a decline in rice production. In the 9-year experiment, the rice grain yield was positively related to soil labile P and slowly cycling P but was unrelated to occluded P in the RFF, RF and RO treatments. Compared with the concentrations of most inorganic P and organic P fractions in the soil in the RO treatments, those in the RF treatment and, in particular, in the RFF treatment increased. This indicated that labile P and slowly cycling P were two important limiting factors for the yield of crops in the subtropical rice paddy fields. The lower soil labile P in the RO system might be related to the lower soil pH, because labile P is rapidly transformed into immobile forms under low pH^31^. The increase in soil organic P (NaHCO_3_-Po and NaOH-Po) may be due to the input of extra P from frog excrement and feed sources. The increase in inorganic P may be due to the reduction in P adsorption and the conversion of Po into Pi in the soil through the mineralization of organic matter. This is corroborated by the findings of Teng^8^, which showed that introducing frogs and fish could also increase soil enzyme activity and microbial biomass. The frogs introduced into RF produced frog dung, which increased the nutrient content and the fungal abundance^32^. On the other hand, the soil P fraction transformation and redistribution in response to three integrated agro-ecological cultures are major controlling factors that increase P solubility and improve the P status of plants. In all, changes in P fractions depended on the net transfer of phosphorus from the mineral soil to the growing vegetation, which was established by the physicochemical and biological properties of soil in the rice paddy fields.

The decline in the concentration of soil total P in the RO treatment over time indicated the continuous depletion of available P due to rice plant uptake over the 9-year period of the integrated agro-ecological cultures. The soil total P fraction was higher in the RFF and RF cultures than that in the RO culture. The likely cause of this pattern may be that the feed for fish and frogs contained P and the unconsumed feed was left in the soil, which then increased soil P in the RFF and RF cultures. However, the annual P input through feed requires further research in the future. The gradual accumulation of labile P and slowly cycling P in rice paddy soils in the RFF and RF cultures may be attributed to several factors. Frogs and fish prey on insects during their aquatic life cycles, which allows nutrient translocation in agro-ecosystems, as part of the dead insects is restored to the underwater ecosystem, resulting in an increase in both labile P and slowly cycling P^33^. The second factor is that frogs and fish absorb all kinds of natural feed sources and excrete excess nutrients into the water, thus accelerating the turnover of organically bound nutrients^34^. Another factor is that dead frogs were not removed from the experimental paddy fields, which allowed fish to decompose their bodies. A fourth reason for the accumulation is that the disturbance of the soil-water interface by fish may result in a release of fixed nutrients from the soil to the water^23^. In addition, it has been shown that long-term application of RFF culture in subtropical paddy fields could improve soil phosphorus availability, which may increase rice productivity.

## Conclusions

This 9-year experiment demonstrated that rice grain yield increased between rice-frog-fish culture and rice-fish culture, while it decreased in the rice-only culture in southeastern China. A deficiency of phosphorus will adversely affect the yield of crops, especially in highly weathered soils. It is interesting to note that the sequential phosphorus fractionation procedure revealed changes in phosphorus fractions in the different farming systems. Soil labile P and slowly cycling P increased with time in the rice-frog-fish culture and rice-fish culture due to the existence of frogs and fish, while they generally decreased in the rice-only culture due to the long-term cultivation of rice plants. The rice-frog-fish farming system led to an improvement in soil phosphorus availability in the long term, which increased the rice grain yield. This study thus concluded that the RFF and RF farming systems were the best practices for improving rice grain yields and soil fertility. Furthermore, these findings provide unique insights into the important role that modern agricultural systems play in contributing to food security. Further research is required on the deficiency of one or more micronutrients (such as Ca, Mg, Cu, Mn, Zn, and B), which affect both the yield and quality of crops in the different farming systems on subtropical rice paddy fields.

## Materials and Methods

### Field experiments

The study was carried out at the Hong-Jian Science and Technology Farm (27°31′22″N, 117°18′22″E) in Guangze County, Fujian, southeastern China, from May 2008 to October 2016. This region is in the subtropical monsoon zone, which is characterized by high temperature and heavy rainfall. The average annual rainfall is 185–220 cm, and the temperature covers a range from a maximum of 39.7 °C in the summer to a minimum of minus 10.8 °C in the winter. The relative humidity is 79%. The soils were derived from a slope deposit of red soil from a small hilly region and were typical yellow clay found most often in humid, warm temperate regions. The soil contained 32.5 g kg^−1^ of organic matter, 2.3 g kg^−1^ of total N, 0.34 g kg^−1^ of total P and 49 g kg^−1^ of total K.

A randomized complete block design with three replications was used for the three treatments, namely, rice-frog-fish (RFF), rice-fish (RF) and rice-only (RO). Each block was set up with three replications on a total of nine plots of land. Each plot had a size of 120 m^2^ and was separated by a levee (40 cm in height), which prevented the irrigated water from leaking out. A ditch full of water was built at the corner of each plot to provide more comfortable habitats for the frogs. Furthermore, the plots were enclosed by 1 m high nylon nets (45 mm mesh size) to prevent piscivorous predators, such as herons, from foraging. The experiment and sampling of the cropping system are shown in Table 4. *Astragalus sinicus* was planted in the plots in the winter, and the fields were plowed three times using a power tiller in full sun for a substantial period. After basic dressing, rice was transplanted using a rake. The rice (*Oryza sativa* L.) seedlings were transplanted from a nursery into the experimental plots 27 days after base seeding. The seedlings were transplanted at a planting density of 20 × 20 cm, with two to three seedlings per hill in the RO plots. The RFF and RF plots had a narrower spacing of 15 cm next to a wider spacing of 30 cm, which allowed the frogs to leap. For the proper management of rice, all activities were carried out according to the recommendations found in Farming System^35^. The tiger frogs (*Rana tigrina*) used in this experiment were a hybrid between pure Thailand tiger frogs and local tiger frogs. These frogs had a low temperature resistance, a short larval stage, a strong ability to catch insects, and fast growth rates and were hardy. Nile tilapia (*Oreochromis niloticus*) were used, as they are well suited for rice-frog-fish farming in a subtropical climate. All the tiger frogs and Nile tilapia were fed according to the breeding standards approved by the National Research Council (NRC). The use and care protocols for the tiger frogs and Nile tilapia were approved by the Laboratory Animal Ethics Committee, Nanping city (20060030012). All efforts were made to minimize the animals’ suffering.

**Table 4:** Experimental and sampling calendar.

Date	Activities
20 December of winter in the previous year	Planting *Astragalus sinicus*
12 March-23 April	Plowed and left to fallow three times
22 April-29 April	Seeded rice in nursery
12 May-14 May	Fertilized field: base dressing
22 May-26 May	Transplanted rice
17 June-20 June	*Tilapia mossambica* (fish) was stocked
24 June-27 June	*Rana tigrina* (frog) was stocked
22 July-28 July	Fertilized field: top-dressing
29 July-5 September	Weeding and pest control
20 September-20 October	Rice was sampled and threshed

The stocking density of the frogs and fish was 50000–80000 individuals per hectare and 1200–2000 individuals per hectare, respectively, according to the local habitats. There were no pesticides applied during the entire experimental period in accordance with the USDA organic standards. Organic fertilizer was applied as the base dressing in all the treatments before transplanting and plowing, and the amounts of N, P, and K were 47.0, 38.1, and 15.8 kg ha^−1^, respectively. Inorganic fertilizer was added as a top-dressing (40.4 kg N ha^−1^ and 20.2 kg K ha^−1^).

### Soil sampling and analysis

To evaluate the responses of phosphorus fractions and phosphorus availability to the rice-frog-fish farming system compared to those to the rice-fish and rice-only farming systems, we used archived soil collections from a 9-year field experiment in several paddy fields in southeastern China. These well-preserved samples from 2008, 2010, 2012, 2014, and 2016 were used in this study for the chemical analysis of soil phosphorus fractionation. The soil samples were collected after the rice crop harvests and sampled from the 0–15 cm soil layer using a 6.8 cm bulk density corer. Then, the soil samples were taken to the laboratory. After removing roots and stones, the soil samples were air-dried, ground, and sieved (<2 mm).

The soil P fractionation method was first described by Hedley *et al*. (1982) and then modified by Crews *et al*.^21^ and Motavalli and Miles^36^. These methods were employed in this study to quantify the soil phosphorus composition, and four sequential P fractions were analyzed: 0.5 M NaHCO_3_-extractable (pH 8.5), 0.1 M NaOH-extractable, 1.0 HCl-extractable and residual. The procedure in this study omitted the first steps described by Hedley *et al*. (1982), which use anion-exchange resin, because it was assumed that resin Pi is included as part of the bicarbonate Pi^17^. We began the procedure from the bicarbonate (NaHCO_3_) stage as “labile” phosphorus. The slowly cycling P is the pool from which phosphorus can be converted relatively easily to labile P^37^. Slowly cycling P was included in the 0.1 NaOH-extractable phosphorus and 1.0 HCl-extractable phosphorus, which can be derived from phosphate radicals that have adsorbed onto (mostly colloidal) soil particles. These P fractions can also be derived from reactions between phosphate radicals and elements such as calcium (Ca^2+^), aluminum (Al^3+^), iron (Fe^3+^), or other more stable forms of organic phosphorus. The occluded (residual) P after sequential extraction was analyzed by using microwave acid digestion/dissolution and was believed to be extremely insoluble inorganic and organic compounds. The concentration of phosphorus in each supernatant was measured colorimetrically with a spectrophotometer at 712 nm according to Murphy and Riley^38^. The total P concentration in all the extracts was determined by ICP-AES. Organic phosphorus was expressed as the concentration of total phosphorus minus the concentration of inorganic phosphorus. The summation of all the soil P sequential extraction steps was conducted according to the specifications of Chen^39^.

### Data analysis

The differences in rice grain yield among the treatments were identified at the p < 0.05 level with one-way ANOVA (least significant difference (LSD)) using SPSS 17.0 software. The experimental data on soil phosphorus fractions were subjected to a normality test before being analyzed by a repeated-measures ANOVA. The significance of the differences in phosphorus fractions was obtained using the paired *t*-test for time and farming systems. In addition, partial correlation coefficients were calculated and graphs were made using SPSS 17.0.
